# Supplementary material for: R-loops and regulatory changes in chronologically ageing fission yeast cells drive non-random patterns of genome rearrangements
Source: PLoS Genet. 2021 Aug 31;17(8):e1009784. doi: 10.1371/journal.pgen.1009784 (PMC8437301; doi:10.1371/journal.pgen.1009784)
Supplement: S1 Fig — Examples #1 and #2 show classic split reads with mapped mates, where a portion of the coloured read in Part 1 does not map anywhere near the read which has been anchored to its location by its mate. A supplementary alignment of the unmapped part shows that it maps to a distal region of the genome (Part 2). Example #3 shows a situation where the mate of the read is not mapped (note that there is no line connecting either of the alignments in Part 1 or 2 to an anchoring read). Example #4 shows a more complex rearrangement between two nearby regions where the green portion (centre of the Part 1 panel, left of Part 2 panel) has been clipped and the supplementary alignment (purple) is mapped nearby in the same orientation. Depending on the relative location of each portion of the read, this type of event is suggestive of a deletion, translocation or repeat expansion/contraction. Note that in all examples, these reads are rare and our pipeline does not use the same levels of support required by most SV callers. Examples are snapshots from IGV. (PDF) [file pgen.1009784.s001.pdf]

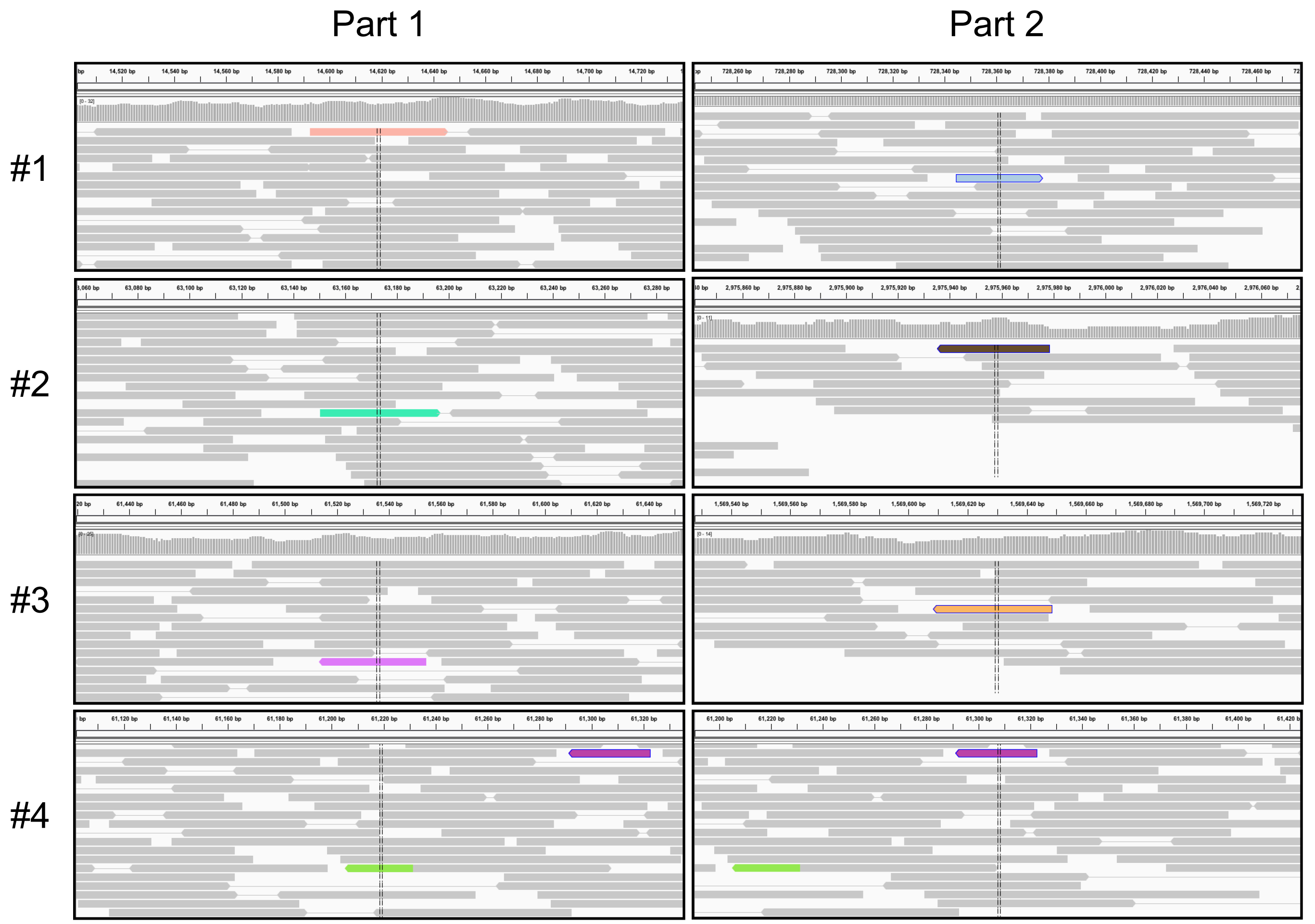

S1 Fig: Examples of split reads. Examples #1 and #2 show classic split reads with mapped mates, where a portion of the coloured read in Part 1 does not map anywhere near the read which has been anchored to its location by its mate. A supplementary alignment of the unmapped part shows that it maps to a distal region of the genome (Part 2). Example #3 shows a situation where the mate of the read is not mapped (note that there is no line connecting either of the alignments in Part 1 or 2 to an anchoring read). Example #4 shows a more complex rearrangement between two nearby regions where the green portion (centre of the Part 1 panel, left of Part 2 panel) has been clipped and the supplementary alignment (purple) is mapped nearby in the same orientation. Depending on the relative location of each portion of the read, this type of event is suggestive of a deletion, translocation or repeat expansion/contraction. Note that in all examples, these reads are rare and our pipeline does not use the same levels of support required by most SV callers. Examples are snapshots from IGV.
