# Supplementary material for: R-loops and regulatory changes in chronologically ageing fission yeast cells drive non-random patterns of genome rearrangements
Source: PLoS Genet. 2021 Aug 31;17(8):e1009784. doi: 10.1371/journal.pgen.1009784 (PMC8437301; doi:10.1371/journal.pgen.1009784)
Supplement: S2 Fig — Raw sequencing reads (126 nt paired-end, Illumina) were aligned to the reference genome with BWA-MEM. Alignment files were then converted to BAM format and sorted before having PCR duplicates removed in Samtools. Next, Bash commands (Awk and Grep) were used to extract split reads from these files. A custom Python script was used to filter these split reads to obtain a robust set of junctions representing two juxtaposed DNA locations, and to characterise their microhomology use across the junction. (PDF) [file pgen.1009784.s002.pdf]

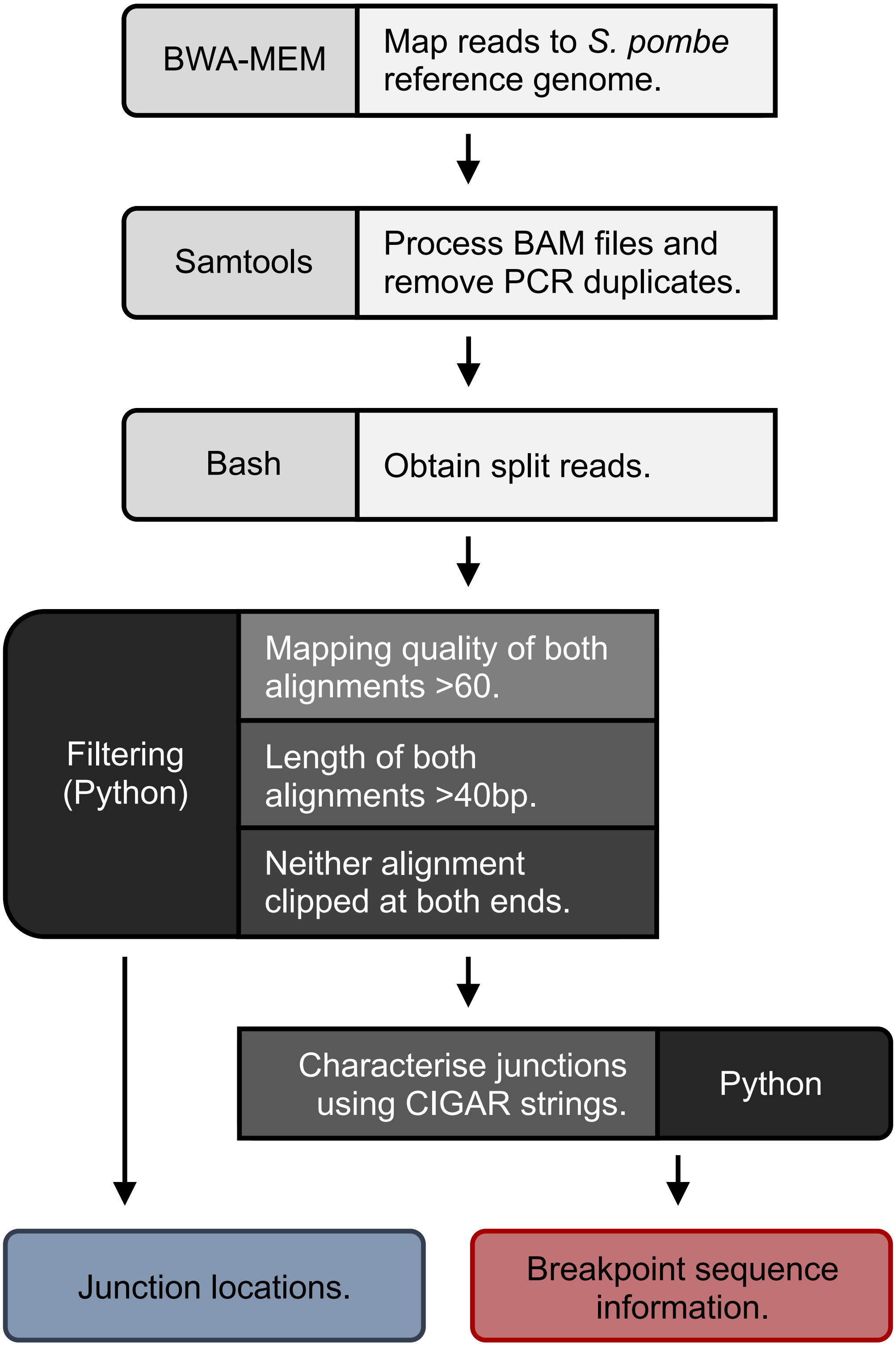

S2 Fig: Pipeline used for identification of breakpoint junctions. Raw sequencing reads (126 nt paired-end, Illumina) were aligned to the reference genome with BWA-MEM. Alignment files were then converted to BAM format and sorted before having PCR duplicates removed in Samtools. Next, Bash commands (Awk and Grep) were used to extract split reads from these files. A custom Python script was used to filter these split reads to obtain a robust set of junctions representing two juxtaposed DNA locations, and to characterise their microhomology use across the junction.
