# Supplementary material for: R-loops and regulatory changes in chronologically ageing fission yeast cells drive non-random patterns of genome rearrangements
Source: PLoS Genet. 2021 Aug 31;17(8):e1009784. doi: 10.1371/journal.pgen.1009784 (PMC8437301; doi:10.1371/journal.pgen.1009784)
Supplement: S3 Fig — Pie charts showing numbers of junctions between mitochondrial and nuclear (green), mitochondrial and mitochondrial (purple), and nuclear and nuclear (orange) DNA in simulated data (left, based on read depth at mitochondrial DNA and nuclear chromosomes) and in measured data (right). (PDF) [file pgen.1009784.s003.pdf]

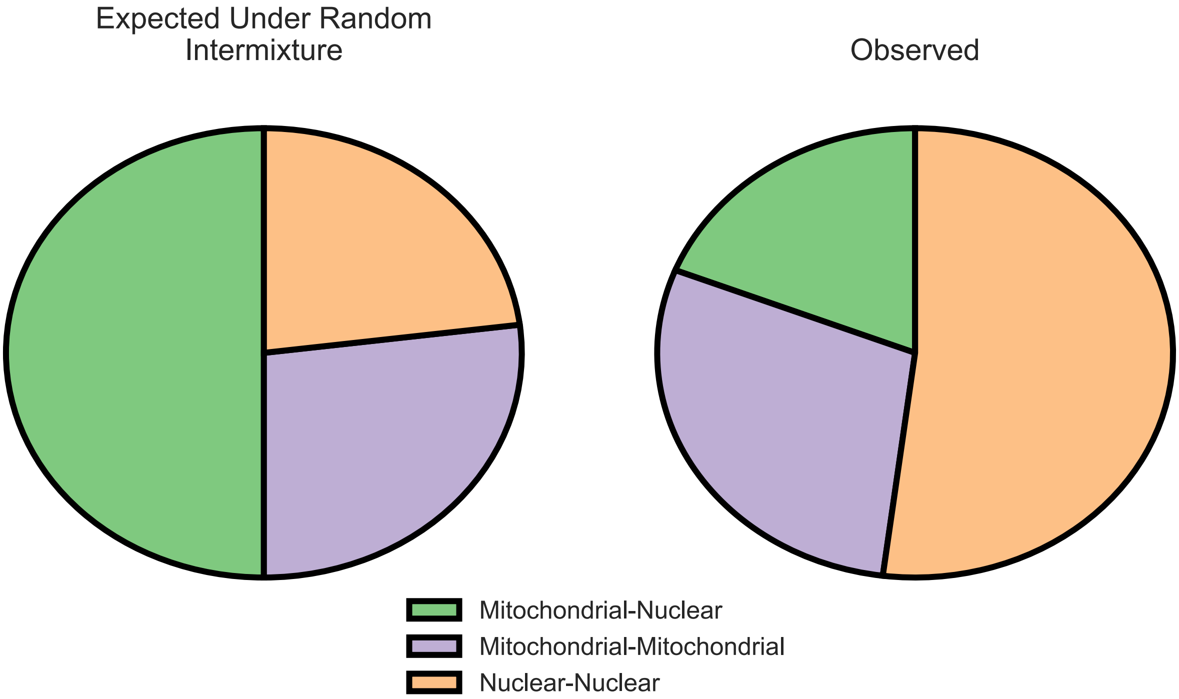

S3 Fig: Proportion of different junction types is not proportional to available DNA during library preparation. Pie charts showing numbers of junctions between mitochondrial and nuclear (green), mitochondrial and mitochondrial (purple), and nuclear and nuclear
(orange) DNA in simulated data (left, based on read depth at mitochondrial DNA and nuclear chromosomes) and in measured data (right).
