# Supplementary material for: R-loops and regulatory changes in chronologically ageing fission yeast cells drive non-random patterns of genome rearrangements
Source: PLoS Genet. 2021 Aug 31;17(8):e1009784. doi: 10.1371/journal.pgen.1009784 (PMC8437301; doi:10.1371/journal.pgen.1009784)
Supplement: S4 Fig — Kernel density and box plots showing the distribution of distances between juxtaposed pieces of DNA for any junction where one juxtaposed piece of DNA is at a global hotspot (bottom; light blue) and all other intra-chromosomal junctions at Day 5 (top; dark blue). These two groups were compared with a two-sample Wilcoxon rank sum test (p<0.0001). (PDF) [file pgen.1009784.s004.pdf]

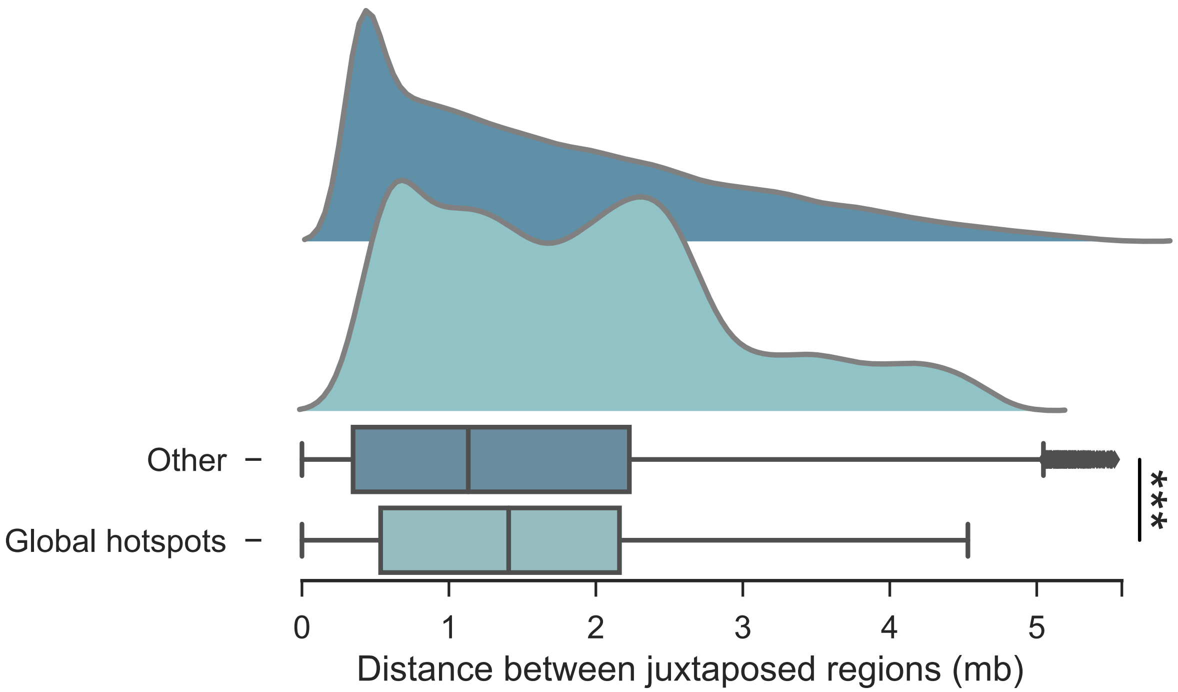

S4 Fig: Local DNA is preferred to distal DNA in intra-chromosomal rearrangements, but less so at global hotspots. Kernel density and box plots showing the distribution of distances between juxtaposed pieces of DNA for any junction where one juxtaposed piece of DNA is at a global hotspot (bottom; light blue) and all other intra-chromosomal junctions at Day 5 (top; dark blue). These two groups were compared with a two-sample Wilcoxon rank sum test ($p<0.0001$).
