# Supplementary material for: R-loops and regulatory changes in chronologically ageing fission yeast cells drive non-random patterns of genome rearrangements
Source: PLoS Genet. 2021 Aug 31;17(8):e1009784. doi: 10.1371/journal.pgen.1009784 (PMC8437301; doi:10.1371/journal.pgen.1009784)
Supplement: S6 Fig — A: Scheme showing how, in a model where repeats do not expand, the number of repeats at a locus should not affect the early:late ratio of junction numbers. B: Bee swarm plot showing the early:late ratio of read depth at each 20kb window, including the hotspots (red). A read depth ratio greater than 0 would indicate an increase in read depth (i.e. repeat expansion). (PDF) [file pgen.1009784.s006.pdf]

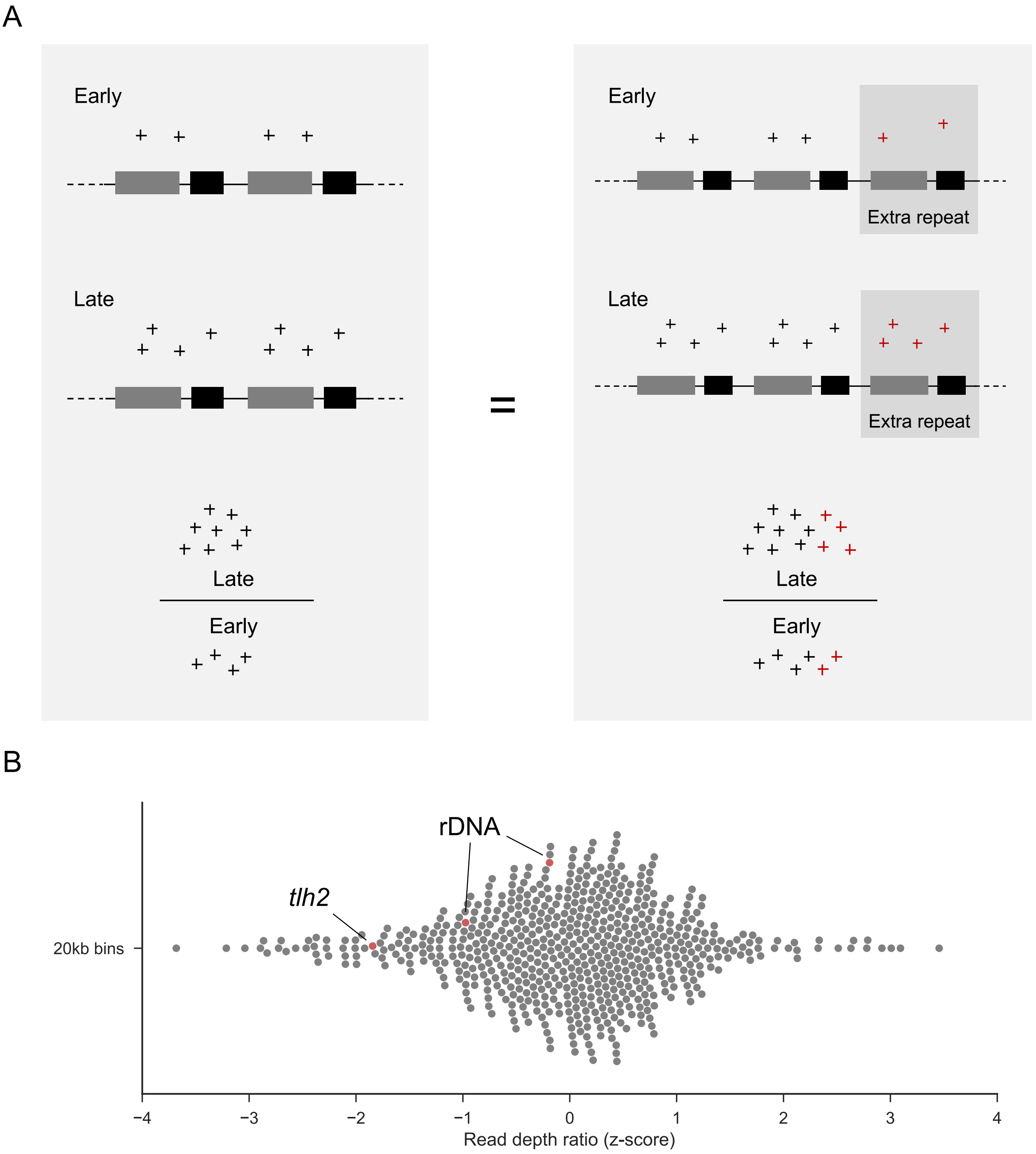

S6 Fig. Repeats are not responsible for extreme junction formation at hotspots.
A: Scheme showing how, in a model where repeats do not expand, the number of repeats at a locus should not affect the early:late ratio of junction numbers.
B: Bee swarm plot showing the early:late ratio of read depth at each 20kb window, including the hotspots (red). A read depth ratio greater than 0 would indicate an increase in read depth (i.e. repeat expansion).
