# Supplementary material for: R-loops and regulatory changes in chronologically ageing fission yeast cells drive non-random patterns of genome rearrangements
Source: PLoS Genet. 2021 Aug 31;17(8):e1009784. doi: 10.1371/journal.pgen.1009784 (PMC8437301; doi:10.1371/journal.pgen.1009784)
Supplement: S7 Fig — A: Left: Reads Per Kilobase Million (RPKM) of tlh2 at the start of the ageing time course (100% cell viability) and when cell viability dropped to 50%. Two biological repeats of the time course were performed (each point corresponds to a repeat). Right: The same data as in Fig 4C are shown on a different scale to reveal the increased tlh2 expression in older cells (50% viability). B: Left: Scheme for calculation of proportion of junctions downstream of tlh2 (grey region; red crosses). Right: Results from three independent repeats with wild type (WT) and tlh2 overexpression cells (tlh2OE). One-sided two sample Mann-Whitney U to test whether tlh2OE is greater than WT (U = 9, p = 0.04). C: Chronological lifespan of tlh2OE (red) compared to WT (grey). Lines show means of three repeats ± 68% confidence intervals. (PDF) [file pgen.1009784.s007.pdf]

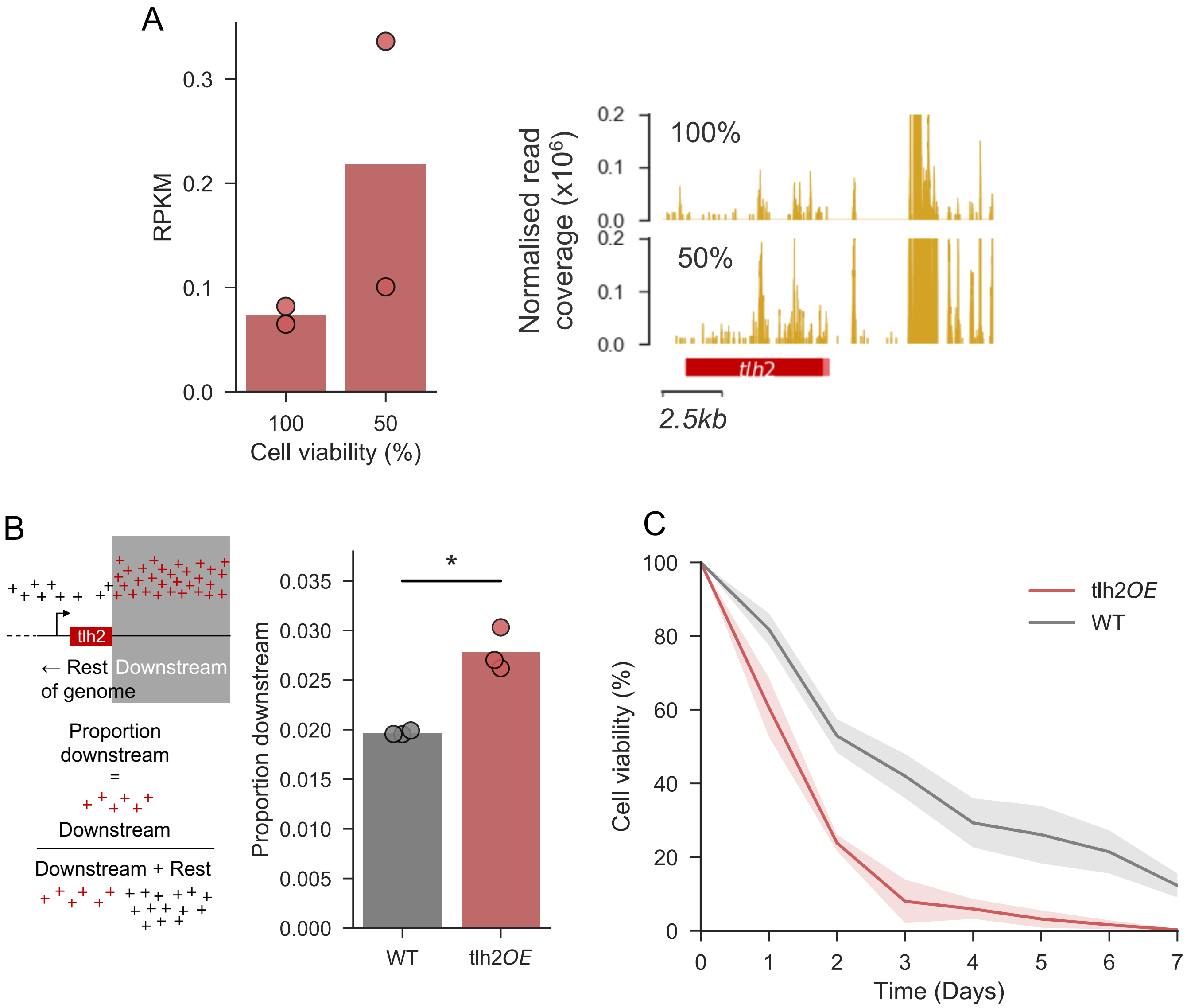

S7 Fig. Transcriptional activity at the tlh2 locus may have a minor role in junction formation at global hotspots.
(A) Left: Reads Per Kilobase Million (RPKM) of tlh2 at the start of the ageing time course (100% cell viability) and when cell viability dropped to 50%. Two biological repeats of the time course were performed (each point corresponds to a repeat). Right: The same data as in Fig. 4C are shown on a different scale to reveal the increased tlh2 expression in older cells (50% viability).
(B) Left: Scheme for calculation of proportion of junctions downstream of tlh2 (grey region; red crosses). Right: Results from three independent repeats with wild type (WT) and tlh2 overexpression cells (tlh2OE). One-sided two sample Mann-Whitney U to test whether tlh2OE is greater than WT ($U=9$, $p=0.04$).
(C) Chronological lifespan of tlh2OE (red) compared to WT (grey). Lines show means of three repeats ± 68% confidence intervals.
