# Supplementary material for: R-loops and regulatory changes in chronologically ageing fission yeast cells drive non-random patterns of genome rearrangements
Source: PLoS Genet. 2021 Aug 31;17(8):e1009784. doi: 10.1371/journal.pgen.1009784 (PMC8437301; doi:10.1371/journal.pgen.1009784)
Supplement: S8 Fig — A: RIP-ChIP data showing log2-fold enrichment in Scw1 pull downs of all canonical target RNAs (81) with similar behaviour in both repeats and data at each time point. Two repeats were sampled at Day 0 (top), Day 2 (middle) and Day 4 (bottom rows). Clusters that show reduced enrichment with age are highlighted in purple. B: Example ChIP-seq of Scw1-TAP. Plots show normalised read depth (mean of two repeats) at three Scw1 targets (red) and three non-targets (blue) in cells aged for two days. For this visualisation, we chose genes which do not overlap any other annotations and matched targets with non-targets of similar gene and 3’ UTR lengths. (PDF) [file pgen.1009784.s008.pdf]

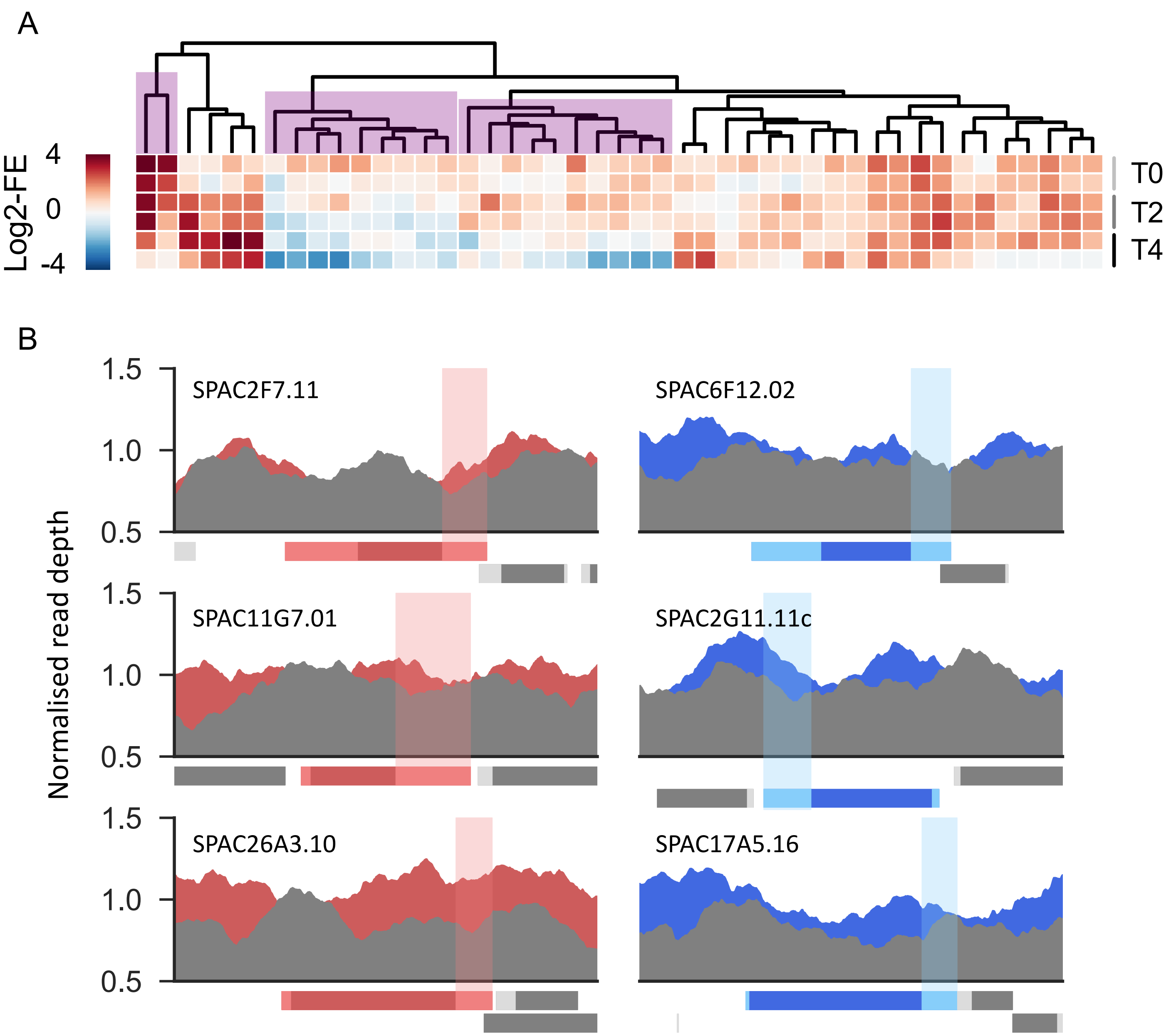

S8 Fig: During ageing, Scw1 binds less to some canonical RNA targets and does not bind to DNA.

A: RIP-ChIP data showing log2-fold enrichment in Scw1 pull downs of all canonical target RNAs (81) with similar behaviour in both repeats and data at each time point. Two repeats were sampled at Day 0 (top), Day 2 (middle) and Day 4 (bottom rows). Clusters that show reduced enrichment with age are highlighted in purple.

B: Example ChIP-seq of Scw1-TAP. Plots show normalised read depth (mean of two repeats) at three Scw1 targets (red) and three non-targets (blue) in cells aged for two days. For this visualisation, we chose genes which do not overlap any other annotations and matched targets with non-targets of similar gene and 3' UTR lengths.
