# Supplementary material for: R-loops and regulatory changes in chronologically ageing fission yeast cells drive non-random patterns of genome rearrangements
Source: PLoS Genet. 2021 Aug 31;17(8):e1009784. doi: 10.1371/journal.pgen.1009784 (PMC8437301; doi:10.1371/journal.pgen.1009784)
Supplement: S9 Fig — A: Representative images of nuclear spreads from wild-type and rnh1Δ rn1201Δ double mutant cells, with the latter known to feature increased R-loop formation [94]. Nuclei are stained with DAPI (blue) and R-loops are detected with the S9.6 antibody (green). To verify that the green signal comes from R-loops, control slides (right hand panels) were treated with the R-loop specific RNAse H before adding the primary antibody. Scale bar 10 μm. B: R-loop signal quantification for nuclear spreads shown in A. Chi-square: p <0.0001, N >50. C: It has been reported that in whole human cells most of the S9.6 signal arises from ribosomal RNA rather than R-loops, and that S9.6 signal remains unchanged by pretreatment with RNase H [95]. Our signal from isolated chromatin, however, was resistant to RNase III but sensitive to RNase H. Representative fields of nuclear spreads from wild-type and scw1Δ cells, each including untreated cells, cells pre-treated with RNase III, and cells pre-treated with RNase H, as indicated. Scale bar 10 μm. (PDF) [file pgen.1009784.s009.pdf]

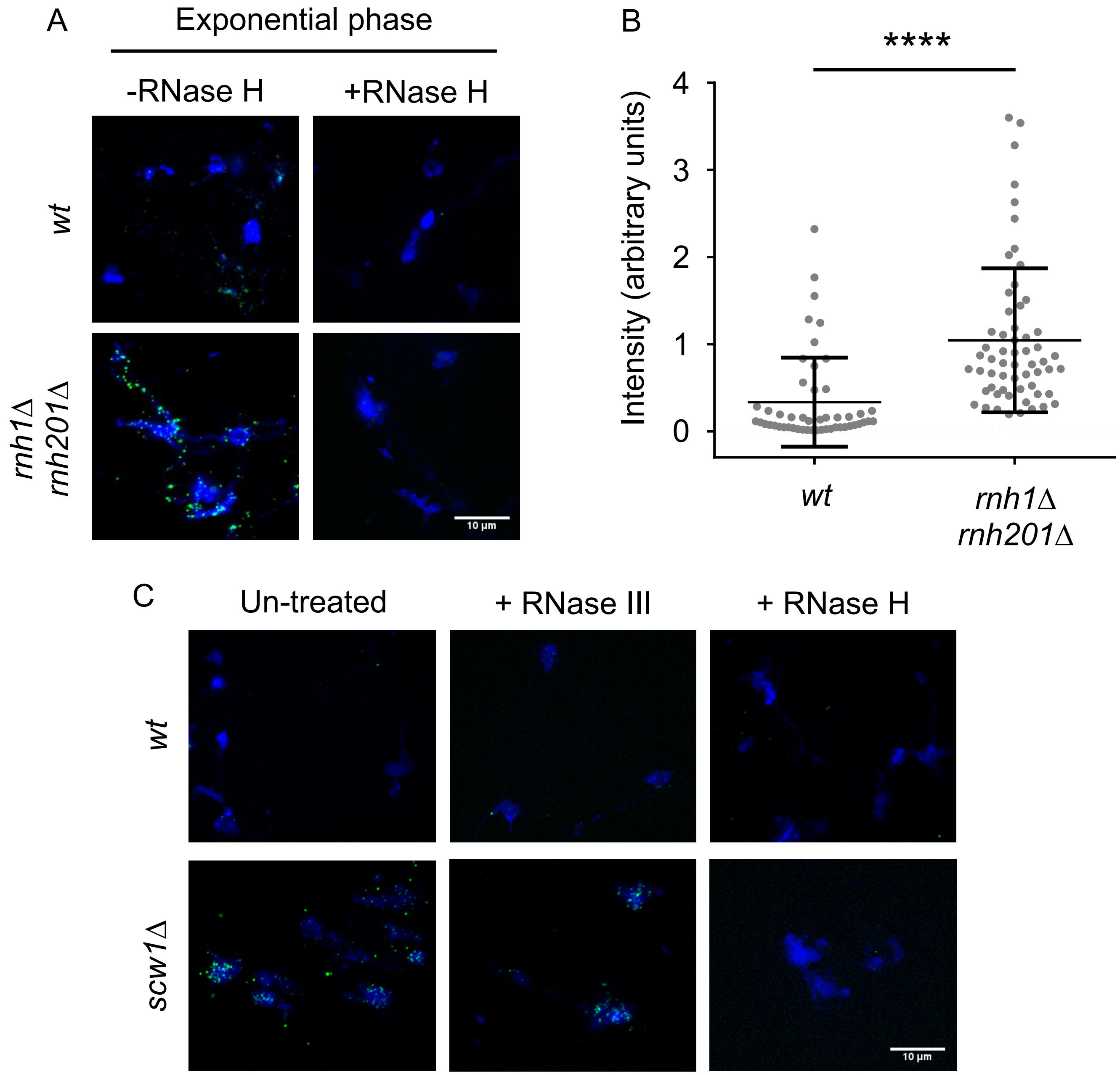

S9 Fig: R-loop immunostaining controls in exponential culture.

A: Representative images of nuclear spreads from wild-type and *rnh1Δ rn1201Δ* double mutant cells, with the latter known to feature increased R-loop formation [95]. Nuclei are stained with DAPI (blue) and R-loops are detected with the S9.6 antibody (green). To verify that the green signal comes from R-loops, control slides (right hand panels) were treated with the R-loop specific RNAse H before adding the primary antibody. Scale bar 10 µm.

B: R-loop signal quantification for nuclear spreads shown in A. Chi-square: $p < 0.0001$, $N > 50$.

C: It has been reported that most of the S9.6 signal derives from RNase T1-and RNase III-sensitive RNA molecules in whole cells [126]. Our signal from isolated chromatin, however, was resistant to RNase III but sensitive to RNase H. Representative fields of nuclear spreads from wild-type and *scw1Δ* cells, each including untreated cells, cells pre-treated with RNase III, and cells pre-treated with RNase H, as indicated. Scale bar 10 µm.
