# Supplementary material for: R-loops and regulatory changes in chronologically ageing fission yeast cells drive non-random patterns of genome rearrangements
Source: PLoS Genet. 2021 Aug 31;17(8):e1009784. doi: 10.1371/journal.pgen.1009784 (PMC8437301; doi:10.1371/journal.pgen.1009784)
Supplement: S10 Fig — In young cells, Scw1 is present and binds its targets at their 3’ UTRs. In old cells, Scw1 is absent and thus leaves nascent RNA of its targets free to anneal with template ssDNA. (PDF) [file pgen.1009784.s010.pdf]

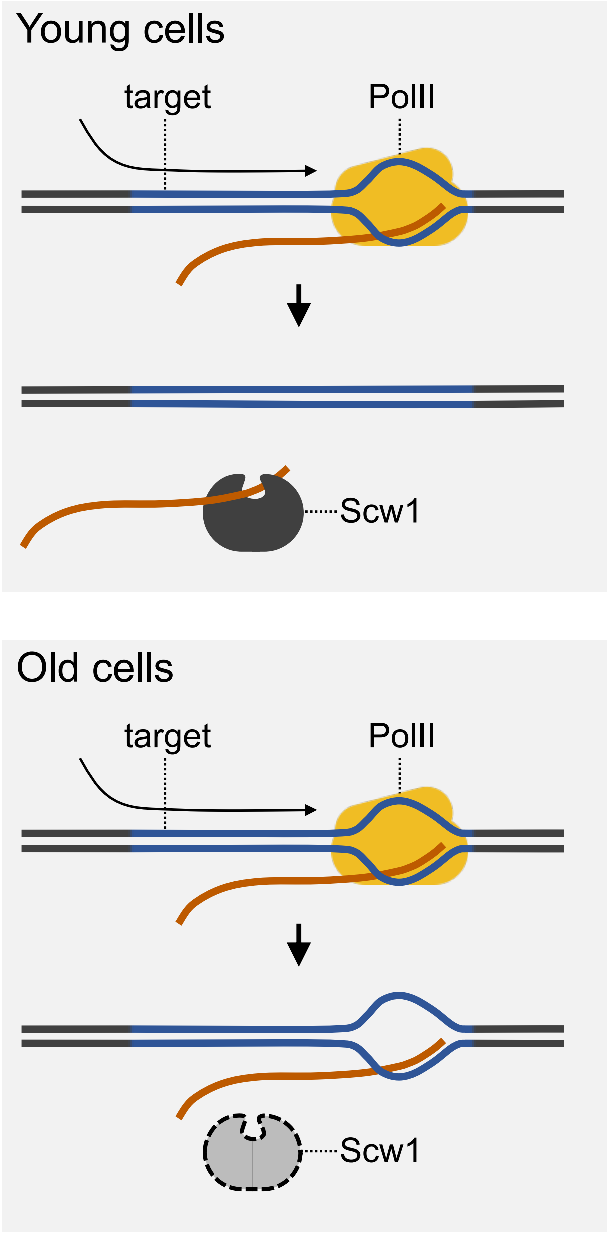

S10 Fig. Model for how Scw1 levels might affect genome rearrangement at its targets. In young cells, Scw1 is present and binds its targets at their 3' UTRs. In old cells, Scw1 is absent and thus leaves nascent RNA of its targets free to anneal with template ssDNA.
