# Supplementary material for: R-loops and regulatory changes in chronologically ageing fission yeast cells drive non-random patterns of genome rearrangements
Source: PLoS Genet. 2021 Aug 31;17(8):e1009784. doi: 10.1371/journal.pgen.1009784 (PMC8437301; doi:10.1371/journal.pgen.1009784)
Supplement: S11 Fig — A: Normalised read depth at all tRNAs in wild-type samples. Blue: non-treated; green: RNase H-treated. B: Region of the S. pombe genome containing a tRNA and rRNA gene. Blue: non-treated; green: RNase H-treated. (PDF) [file pgen.1009784.s011.pdf]

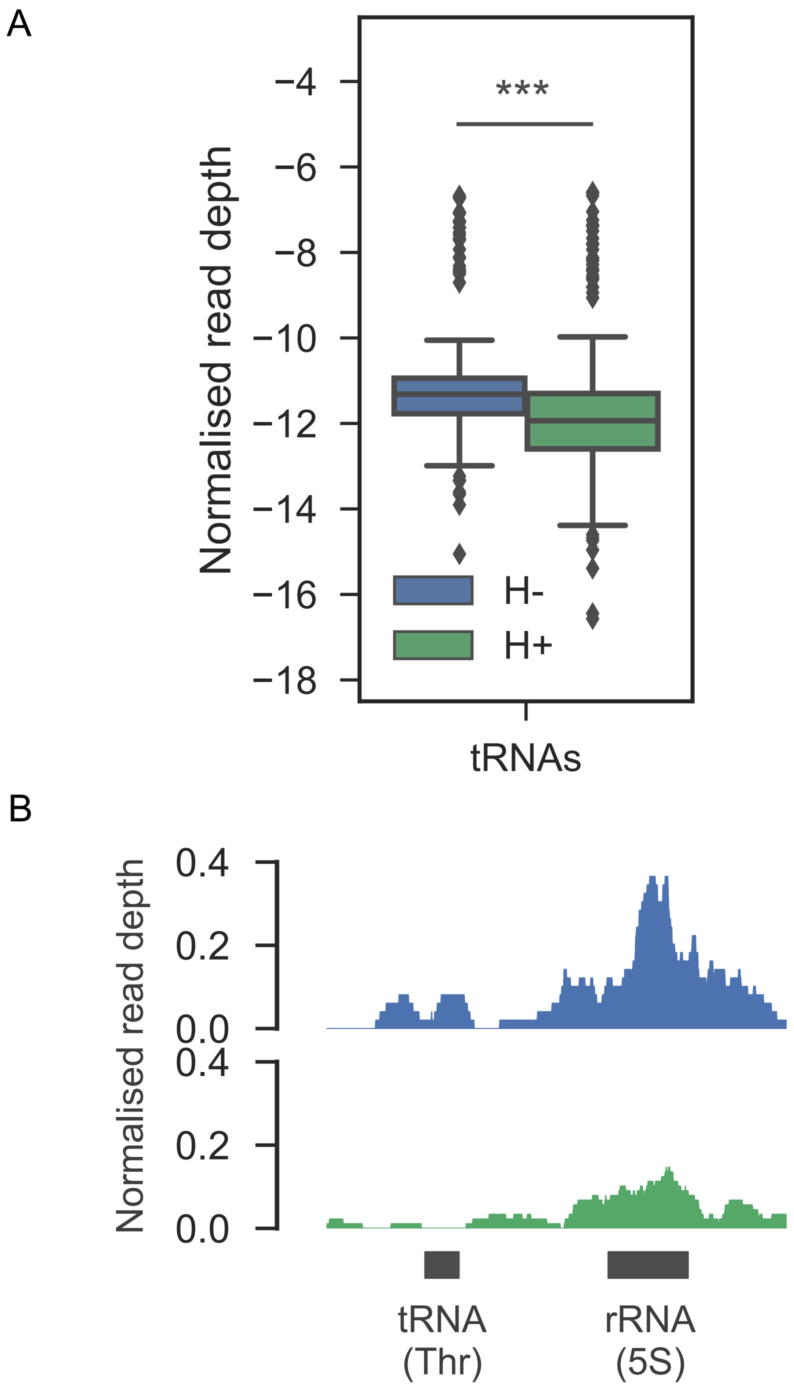

S11 Fig: ChIP of S9.6 produces fewer reads at regions implicated in R-loop formation in RNase H-treated samples.

A: Normalised read depth at all tRNAs in wild-type cells. Blue: non-treated; green: RNase H-treated.
B: Region of the S. pombe genome containing a tRNA and rRNA gene. Blue: non-treated; green: RNase H-treated.
