# Supplementary material for: R-loops and regulatory changes in chronologically ageing fission yeast cells drive non-random patterns of genome rearrangements
Source: PLoS Genet. 2021 Aug 31;17(8):e1009784. doi: 10.1371/journal.pgen.1009784 (PMC8437301; doi:10.1371/journal.pgen.1009784)
Supplement: S1 Text — (PDF) [file pgen.1009784.s012.pdf]

S1 Text

Arguments against breakpoint junctions forming *in vitro.*

In addition to the selection against junctions in coding sequences (Fig 1B), three pieces of evidence argue against the formation of these junctions *in vitro* after DNA extraction. First, during sequence library preparation, dA nucleotides were incorporated at the 3' ends of DNA fragments before ligation of the sequencing adapters (with complementary dT overhangs). We, therefore, expected that false positive concatemers occurring *in vitro* during sequence library preparation should feature insertion of an A at the breakpoint. On the other hand, genuine junctions may feature different signatures at the breakpoint, depending on how they were repaired *in vivo.* Whilst we found a population of single-base insertions (of which A or T insertions could reflect dA-tailing during library preparation), these junctions did not increase at all with age, and age-associated junction formation was associated with microhomology at the breakpoint (Fig 1D). Second, modelling, based on the read depth for each body of DNA (e.g., average depth at Chromosome II), showed that the number of junctions between each type of event (e.g., mtDNA-Chromosome I) was not proportional to the relative number of free DNA ends in the library, with far fewer junctions between mitochondrial DNA and nuclear DNA than would be expected (S3 Fig). Third, the ratio of junction counts between old and young cells was higher for junctions that formed within chromosomes than those that formed between chromosomes (Fig 3B), showing that age-associated junction formation was more prevalent within chromosomes. Furthermore, of those junctions that formed within chromosomes, nearby DNA was preferred to distant DNA (S4 Fig). This pattern echoes recent findings in cancer genomes where the frequency of junction formation between two pieces of DNA is approximately inversely proportional to the distance between them [54].
