# Supplementary material for: R-loops and regulatory changes in chronologically ageing fission yeast cells drive non-random patterns of genome rearrangements
Source: PLoS Genet. 2021 Aug 31;17(8):e1009784. doi: 10.1371/journal.pgen.1009784 (PMC8437301; doi:10.1371/journal.pgen.1009784)
Supplement: S5 Fig — A: All motifs discovered by MEME with e-values <0.05. This analysis was performed on all regions with microhomology-mediated junctions. Regions which aligned with known core sequence motifs of DNA-binding proteins in S. cerevisiae and S. pombe are underlined in grey and black, respectively (see B). B: Table showing the top hits for known S. cerevisiae (grey rows) and S. pombe (blank rows) DNA-binding protein motifs that align to the discovered motifs in A. An ID of the long motif from part A is detailed in the ID column, with the shorter aligning motif in the motif column. (PDF) [file pgen.1009784.s005.pdf]

A

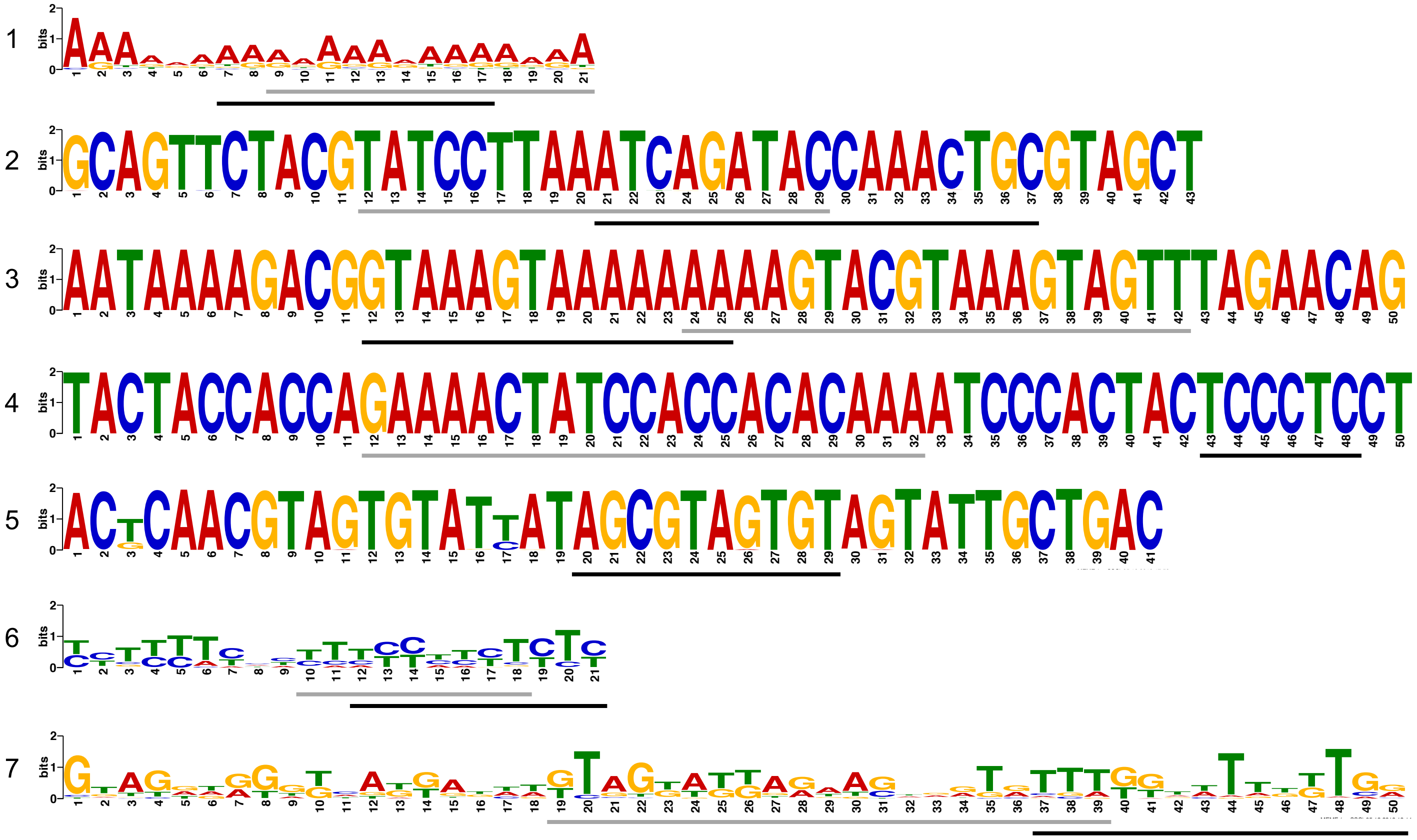

B

ID	factor	description	motif	p-value	e-value
1	ScAzf1	glucose signalling	aAAAAGAAa	<0.0001	<0.05
1	SpSte11	nitrogen starvation	TTCTTTGTTY	<0.01	0.2
2	ScOaf1	telomere silencing	atttCGGatWTAagxxxa	<0.001	0.7
3	ScFhl1	forkhead-like; ribosomal transcription	aaaDxGACGCAaWtxxgtt	<0.01	1.1
3	SpFkh2	forkhead-like; meiotic cell cycle	GTAAACAAACAAAM	<0.01	0.2
4	ScRdr1	multidrug resistance	txxgxcxtGCGGAWAtxxxxc	<0.005	0.9
4	SpRst2	glucose starvation	CCCTC	0.06	2.1
5	SpFhl1	forkhead-like	ACCCTACCCT	<0.01	0.2
6	ScAzf1	glucose signalling	aAAAAGAAa	<0.001	0.6
6	SpSte11	nitrogen starvation	TTCTTTGTTY	<0.01	0.2
7	ScSfl1	stress response; flocculation	tHxHDxataGAAGaaataWDW	<0.05	1.7
7	SpFkh2	forkhead-like; meiotic cell cycle	GTAAACAAACAAAM	<0.05	0.6

S5 Fig: Motifs at microhomology-mediated junctions.
A: All motifs discovered by MEME with e-values <0.05. This analysis was performed on all regions with microhomology-mediated junctions. Regions which aligned with known core sequence motifs of DNA-binding proteins in S. cerevisiae and S. pombe underlined in grey and black, respectively (see B). B: Table showing the top hits for known S. cerevisiae (grey rows) and S. pombe (blank rows) DNA-binding protein motifs that align to the discovered motifs in A. An ID of the long motif from part A is detailed in the ID column, with the shorter aligning motif in the motif column.
