# Supplementary material for: R-loops and regulatory changes in chronologically ageing fission yeast cells drive non-random patterns of genome rearrangements
Source: PLoS Genet. 2021 Aug 31;17(8):e1009784. doi: 10.1371/journal.pgen.1009784 (PMC8437301; doi:10.1371/journal.pgen.1009784)
Supplement: Attachment — Submitted filename: 109414_1_rebuttal_1982587_qny6cz.pdf [file pgen.1009784.s015.pdf]

Response to Reviewers

We appreciate the interest shown by the two reviewers for our manuscript and thank them for their constructive feedback. Below, we provide a point-by-point response to the issues raised, describing the changes we have made and additional experiments we could realistically perform for a revised manuscript within about 2 months.

Reviewer #1:

*****Major points:*****

1. The authors should first demonstrate that their DRIP protocol is robust and reliable. Considering that it is widely accepted that R-loops are fragile structures sensitive to mechanical stress, the use of both a Fast-prep instrument to extract genomic DNA and of sonication to shear the DNA might interfere with the optimal recovery of RNA-DNA hybrids. The authors should therefore demonstrate using DRIP-qPCR that they can efficiently purify RNA-DNA hybrids over positive and negative control regions. They could for example use the rDNA (5' of the 18S) or some ribosomal protein genes as positive controls. For this, they need to show the raw data (% IP) and the RNase H-treated control and it would be advisable for them to show that their DRIP-qPCR signals are indeed stronger when RNase H enzymes are missing (rnh1Δrnh201Δ), as predicted. In rnh1Δrnh201Δ cells, R-loops forming over tRNA genes should be greatly stabilized and this is a convenient test to demonstrate the quality of a DRIP-qPCR experiment.

Reply: Given the strong cell wall of yeast, it was necessary to break them by Fastprep as is standard for such cells. We had also tried various enzymes (lallzyme, zymolyase, lyticase) to break the cells, but these attempts did not produce good results. We do not consider the use of Fastprep a problem given its widespread use in other applications to analyse chromatin (e.g. for ChIP-seq): even if the chromatin were a bit broken, the average fragment size would remain bigger than after sonication. Sonication is an established method to shear the DNA for DRIP [e.g., Ginno et al. *Mol Cell* (2012) 45:814; Wahba et al. *Genes & Dev* (2016) 30:1327; Tan-Wong et al., *Mol Cell* (2019) 76:600] and has been reported to lead to better results than restriction-enzyme digestion [Halász et al. *Genome Res* (2017) 27:1063]. We now cite this information in the revised version of the manuscript.

Before sonication, we treated the samples with S1 nuclease (S1-DRIP), which has been shown to prevent displacement of R-loops during sonication [Wahba et al. *Genes & Dev* (2016) 30:1327]. As a control, we had performed the DRIP-seq protocol with the samples treated by RNase H in parallel. The results showed that RNase H leads to reduced DRIP signals across the tRNA and rRNA genes. We have now added these controls to the revised manuscript (**Fig. S10** below).

We have limited experience with DRIP, and in an ideal situation the difference between RNase H treated and untreated samples should possibly be larger. All things considered, however, we are confident that our DRIP-seq experiment is reliable in capturing R-loops. The proposed experiments would take us at least 3 months to complete under the current restrictions owing to the pandemic. Please note that the DRIP-seq data are not that critical for our paper as they are only used to support independent evidence from other approaches. We cautiously discuss these data in the manuscript.

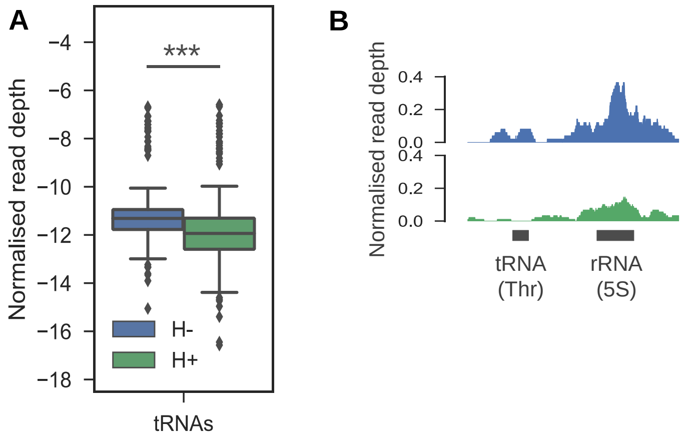

Figure S10. ChIP of S9.6 produces fewer reads at regions implicated in R-loop formation in RNase H-treated samples. A: Normalised read depth at all tRNAs in wild-type samples. Blue: non-treated; green: RNase H-treated. **B:** Region of the S. pombe genome containing a tRNA and rRNA gene. Blue: non-treated; green: RNase H-treated.

2. It is unclear how the authors could have built sequencing libraries from their DRIP material. Considering that they have digested the single stranded part of the R-loop with nuclease S1 (which disappears anyway with sonication), they should have recovered only RNA-DNA hybrids and it is unclear how they have built sequencing libraries from a single strand of DNA (they should refer themselves to publications from the Sun, Chédin and Cimprich labs on this matter). To demonstrate the reliability of their DRIP-seq experiments, the authors must show snapshots of control regions (as in #1) and add both an Input and an RNase H-treated control (Figure 4C and 5E).

Reply: We applied an established method to generate the sequencing library that has been used in multiple papers reporting DRIP results [e.g., Fig. S1 in Wahba et al. *Genes & Dev* (2016) 30:1327; Halász et al. *Genome Res* (2017) 27:1063]. In these samples, not all the DNA is single stranded: for DNA fragments that are long enough, after immunoprecipitation and RNA removal, the fragment ends either side of where the RNA was previously attached will be double-stranded DNA which is sufficient to make the libraries. Given that this is an established protocol, it does not seem sensible to develop and optimize a different protocol for any minor expected gain in the context of our study. As described above, we already have an RNase H-treated control (Fig. 5E), and it would not be feasible to repeat all experiments with the additional tests in a tractable timeframe.

3. They also need to show the size of their DNA fragments after shearing to give an estimate of the resolution of their sequencing experiment. For example, in Fig 4C, how do the authors explain that their DRIP-seq peak is much larger than their RNA-seq peak? Their DRIP-seq peak is in fact surprisingly huge (>5kb); is it because their resolution is poor? The authors should generally be more precise regarding the protocol that they have used (how many units of nuclease S1, what buffer, what instrument/parameters were used for the sonication, amount of DNA, amount of antibody, etc...).

Reply: From our Bioanalyzer trace data (see figure below), we know that the fragments were about 400 bp after sonication, and sequencing libraries were selected to be 250-600 bp. The broad DRIP-seq signal in Fig. 4C can be explained by the presence of multiple R-loops downstream of *tlh2*, which is consistent with the data showing neighbouring peaks rather than a single broad peak. For DRIP-

seq, we used the protocol as published in the cited papers, with the alterations as specified. We now provide these specific details requested by the reviewer in the revised manuscript.

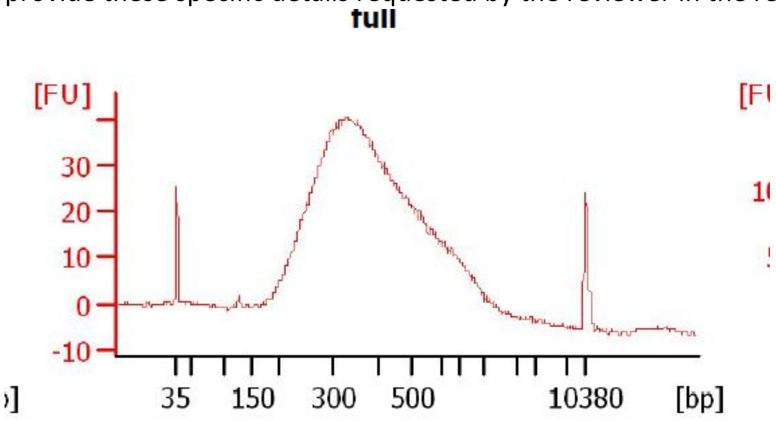

4. Chromosome spreads are not a reliable way of quantifying R-loops, as shown by the fact that most of the S9.6 signal does not actually overlap with DAPI (Fig 5D and S8). Consistent with this, the Chédin lab recently demonstrated that most of the S9.6 signal in IF experiments derives from RNase T1- and RNase III-sensitive RNA molecules (see doi: https://doi.org/10.1101/2020.01.11.902981). The authors should show that their sensitivity to RNase H is specific and that their signal is indeed resistant to RNase T1 or RNase III. This is also why it is so important that the authors should back their observations with reliable DRIP experiments.

Reply: R-loop detection by antibody in nuclear spreads is a well-established approach that has been corroborated by orthogonal experiments and is widely used in the scientific literature [e.g., Wahba et al. *Mol Cell* (2011) 44:978]. Most, if not all, of the S9.6 signal co-localized with DAPI as is evident with increased magnification and brightness to reveal fainter DAPI signals. For example, the picture below shows an enlarged and brighter version of Figure 5D (top left frame), revealing that all green signal co-localizes with chromatin fibres (blue). We now explain this in the manuscript.

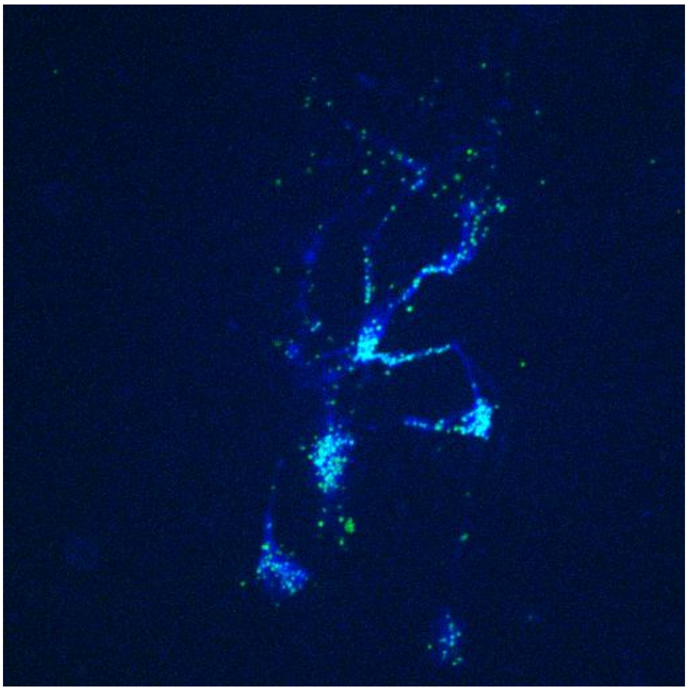

We stress again that non-dividing, ageing cells cannot be broken by gentle enzymatic treatment as is possible with proliferating cells. This is why we used Fastprep treatment to break the cells. The nuclear spreads of aged cells, therefore, show more dispersed chromatin leading to weak DAPI

staining. We tested whether the signal is specific by showing that it disappears upon RNase H treatment, and it is also missing in wild-type controls.

We were not aware of the recent paper by the Chédin lab which has not yet been peer-reviewed. We will cite this preprint in the revised manuscript. Note, however, the data in this preprint refer to whole cells, while our results are based on isolated chromatin. With respect to the controls requested by the reviewer, we actually did test for resistance to RNase III as shown in the figure below. Almost all of the signal was resistant to RNase III but sensitive to RNase H. Below, we show representative fields of nuclear spreads from wild-type and *scw1Δ* cells, each including untreated cells, cells pre-treated with RNase III (showing that the S9.6 antibody signal remains), and cells pre-treated with RNase H (showing that the antibody signal is no longer detected). The same method was used as for Figures 5D and S8. We have added these control data as a supplementary figure in the revised manuscript (Fig. S9C).

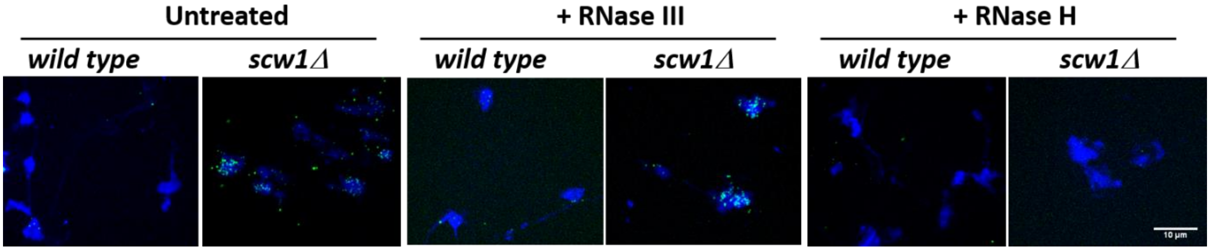

5. It is unclear why the authors have decided to perform their DRIP experiments on exponentially growing cells. For their purpose, it would make a lot more sense to compare DRIP signals at various time of the ageing process, using normalized DRIP-seq data (see for example PMID: 32544226).

Reply: We agree in principle that it would be ideal to perform the DRIP experiments in ageing cells and use a quantitative protocol to study changes in R-loops as a function of age. The paper highlighted by the reviewer is for mammalian cells, however, and it would not be straightforward to implement the approach for yeast cells. In fact, we had tried to establish DRIP analyses in ageing yeast cells, using both mechanical cell breaking or treatment with various combinations and concentrations of enzymes. Unfortunately, these cells become smaller and acquire cell walls that are highly resistant to breaking or digestion. Although our laboratory managed to establish breakage protocols that work for RNA-seq and ChIP-seq in old yeast cells, these methods were not suitable for DRIP-seq, likely because they are too harsh for the fragile R-loops. The reviewer is already concerned by the treatment required to break proliferating cells, and ageing cells would require even harsher treatments that were simply not compatible with DRIP in our attempts. We point out again that the DRIP data are only complementary and not central to our study. In all likelihood, a refined method could eventually be developed to apply DRIP-seq to ageing cells, but this could take one year or more and is not realistic for the timeline of our project.

6. Fig S6A and Fig 4B seem to contradict each other because the subtle increase in tlh2 expression during ageing detected by RNA-seq is not detected by smFISH. Similarly, the results of Fig S6A are not substantiated by the snapshots presented in Fig 4C, which show no significant increase in read density over the tlh2 gene during ageing. This is confusing and the results of Fig S6A should therefore be confirmed by RT-qPCR experiments in triplicates.

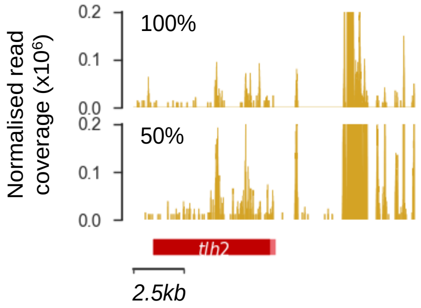

Reply: The subtle increase in *tlh2* expression from bulk RNA-seq data is not evident in Fig 4C, because *tlh2* expression is over-powered by the much stronger expression downstream of *tlh2.* At right, we show the same data on a different scale, which does reveal the increased expression in the body of the *tlh2* gene in older cells (50% viability). We have added this visualization to Fig. S7A.

As discussed in the manuscript, this small expression difference is likely not sufficient to explain the rearrangements. Therefore, we have performed additional experiments, including *tlh2* overexpression and smFISH. We show that overexpression of *tlh2* is sufficient to trigger breakpoint junctions (Fig. S7B) and decrease lifespan (Fig. S7C). The hypothesis that heterogeneous expression between cells was masked in the bulk RNA-seq data was not confirmed by smFISH, as explained in the text. This result then led us to postulate that instead of *tlh2* expression itself, the hotspot might involve Sir2-dependent chromatin alterations and R-loop formation in the downstream region, as suggested by data in Fig. 4B and 4C. Nevertheless, *tlh2* is the nearest gene to this hotspot and is implicated both in age-associated disease and DNA repair, raising intriguing implications. The phenotypes in a transgenic line overexpressing *tlh2* could be due to alteration of downstream chromatin or to an R-loop independent function of the Tlh2 protein. In the revised manuscript, we have clarified and better discussed these results.

In order to obtain more insight into the role of Sir2 to generate rearrangements, we are determining the lifespan of *sir2* deletion mutants and will sequence PCR amplicons of the *tlh2* region in the old *sir2* mutant and wild-type control cells. If successful, these experiments would show whether Sir2 is required for rearrangements in selected hotspots.

7. Fig 4C suggests that transcription is altered downstream of tlh2 rather than within tlh2 during the ageing process. It is unclear how this relates to the results presented in Fig S6B/C, where the transcription of tlh2 itself was manipulated. Did the authors ascertain whether to express tlh2 under the control of the nmt1 promoter also alters transcription downstream of tlh2? How do the authors explain that tlh2OE stimulates rearrangements downstream of tlh2 like in a normal ageing process? Can they detect read-through transcripts and downstream R-loops when they over-express tlh2 or when they delete Sir2?

Reply: Yes, our findings are most consistent with changes in chromatin and transcription downstream of *tlh2* being causal for the rearrangement hotspot as discussed above. We are not sure why *tlh2* overexpression in proliferating cells is sufficient to stimulate rearrangements, but this is a stark, intriguing result. As the reviewer suggests, this points to a link between *tlh2* transcription and downstream chromatin which could either occur naturally during ageing or might be an artefact of transcription triggered by the strong *nmt1* promoter. Although the suggested follow-up experiments are interesting, *tlh2* does not appear to be the sole, or even main cause of rearrangements in the hotspot area based on our other experiments shown. In the revised manuscript, we have clarified the discussion and conclusion in this respect. We have not established a conclusive mechanistic process explaining the rearrangements, and we do not claim in the manuscript that we do. Our findings point to different mechanisms acting at different types of hotspots and suggest that R-loops play a prominent role. More work will be required to flesh out the precise mechanisms. As described above, the additional experiment with the *sir2* deletion mutant might link Sir2 function to rearrangements.

8. If, as suggested by Fig 4C, transcription is altered downstream of tlh2 during ageing, smFISH should be used to confirm this result, using probes downstream of tlh2 overlapping with the RNA-seq signal.

Reply: This experiment seems to be unnecessary given that we see such a strong, local increase in bulk RNA-seq. It is not clear what smFISH would add to the picture. Our collaborator for smFISH, Samuel Marguerat, is not currently in a position to carry out such experiments.

9. The authors say that 148 genes are more likely than expected to have rearrangements in their 3' UTR. Of these, only 17 are targets of Scw1 (out of 227 Scw1 targets). This means that 210 Scw1 targets are not particularly prone to rearrangements, even if they might see a significant increase in R-loop formation in the absence of Scw1 according to the unsatisfactory DRIP-seq experiment (Fig 5E). At the very least, these observations demonstrate that R-loops are not sufficient to trigger rearrangements. What is the evidence presented here that R-loops are necessary to trigger rearrangements?

Reply: We do not claim in the manuscript that R-loops and Scw1 are the sole cause of genome rearrangements. We discuss the different types of hotspots identified. In the specific analysis the reviewer refers to, over 10% of the 148 genes were Scw1 targets. The genes associated with rearrangements are significantly over-represented among the Scw1 targets because Scw1 targets less than 5% of all *S. pombe* genes (227/5133). Moreover, the DRIP-seq results do indicate that R-loops are increased across most or all Scw1 targets, because R-loops increased in the absence of Scw1 even when the 17 Scw1 targets found in the previous analysis were not included in our comparison (one sample t-test: T=-2.46, p=0.014). The DRIP-seq experiment may not be perfect, but it is hard to imagine that such a result would be obtained by accident. Although these results suggest that mechanisms other than R-loops contribute to the rearrangements, we do not understand how they would show that R-loops are not sufficient to trigger some rearrangements as suggested by the reviewer. Our evidence that R-loops are necessary to trigger rearrangements is circumstantial at this point. In order to obtain data showing that R-loops are necessary for the rearrangements, we are determining the lifespan of cells lacking RNase H activity (*rnh1Δ rnh201Δ*) and will sequence PCR amplicons of selected Scw1 target genes in old mutant and wild-type control cells. If successful, these experiments could be added to the paper.

10. The drawing on Fig 5F is misleading as it suggests that R-loops are the result of the hybridization of the nascent RNA within the active site of the RNA polymerase, whilst this is not the case. In addition, there are a lot of proteins binding to the 3' end of the nascent RNA and it is surprising that the loss of Scw1 would be sufficient to expose the 3' end and allow it to reanneal with its template. This seems a little bit naïve. If Scw1 was a master organizer of the 3' end, presumably there would be transcription termination defects in the absence of Scw1 or during ageing. Is this the case?

Reply: We do not quite understand what the reviewer finds misleading in this scheme. Is the problem that the RNA would need to re-invade the DNA strand after having been released by Pol II? Or that Pol II could backtrack (as discussed in Zatreanu et al. 2019, *Mol. Cell*)? This is just a working model at this time, and it is clear that many parts of the puzzle are still missing. We do not know whether *scw1* mutants or ageing cells show any transcription termination effects.

11. To substantiate their claims that R-loops contribute to genome instability during ageing, the authors should investigate whether the ageing process and the number of rearrangements are

altered by manipulating R-loops directly, either by deleting RNase H (rnh1Δrnh201Δ) or by over-expressing RNase H1. In rnh1Δrnh201Δ cells, do they detect more breakpoints specifically where R-loops are stabilized?

Reply: As described in response to Comment 9 above, we are determining the lifespan of cells lacking RNase H activity (*rnh1Δ rnh201Δ*) and will sequence PCR amplicons of selected Scw1 target genes in mutant and wild-type control cells during ageing. We also have a strain overexpressing RNase H, although analysis for this may be confounded owing to the need to use a different growth medium which alters chronological ageing. These are somewhat tricky experiments and we cannot be certain to obtain meaningful results within a realistic timeframe.

*****Minor points:*****
1. It would be helpful to show some examples of "rearranged" reads, carefully explaining their structure to the reader.

Reply: Below we provide a few examples, which we have added as a new Fig. S1 in the revised manuscript.

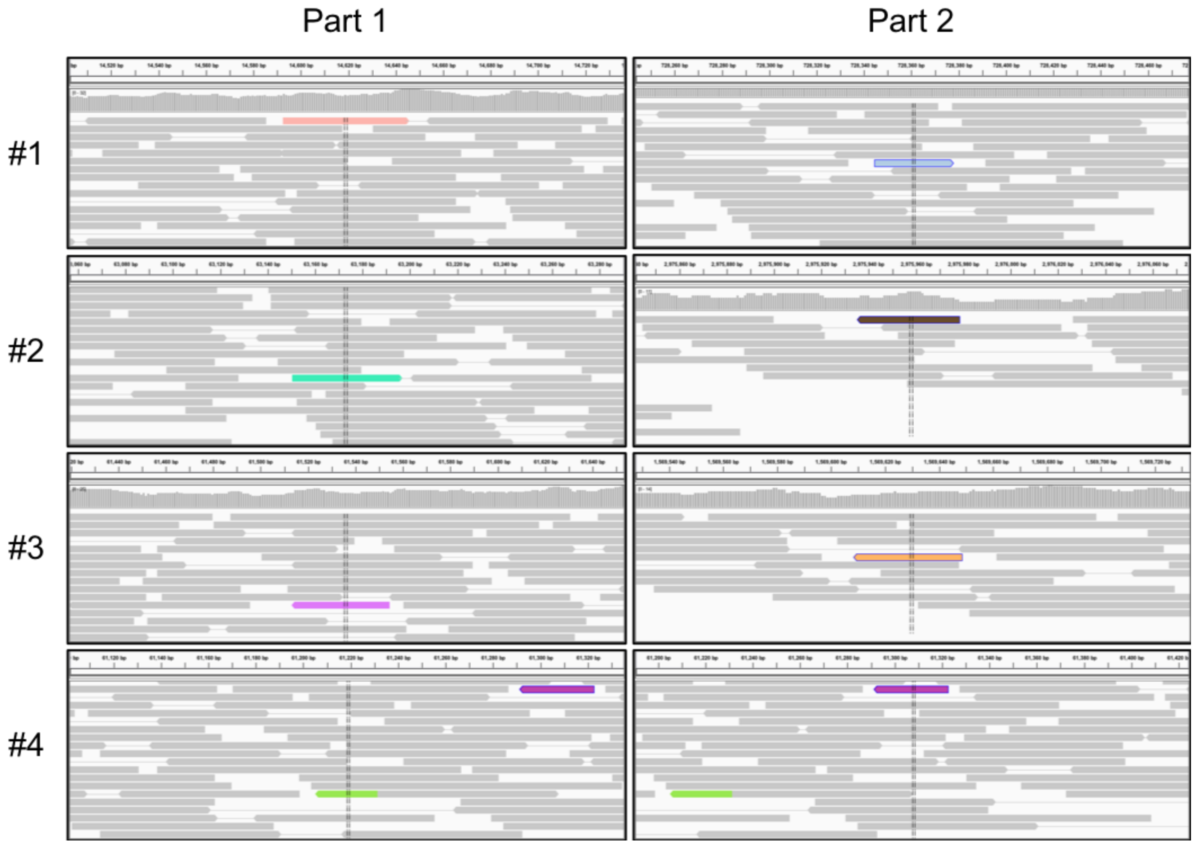

Figure S1. Examples of split reads. Examples #1 and #2 show classic split reads with mapped mates, where a portion of the coloured read in Part 1 does not map anywhere near the read which has been anchored to its location by its mate. A supplementary alignment of the unmapped part shows that it maps to a distal region of the genome (Part 2). Example #3 shows a situation where the mate of the read is not mapped (note that there is no line connecting either of the alignments in Part 1 or 2 to an anchoring read). Example #4 shows a more complex rearrangement between two nearby regions where the green portion (centre of the Part 1 panel, left of Part 2 panel) has been clipped and the supplementary alignment (purple) is mapped nearby in the same orientation. Depending on the relative location of each portion of the read, this type of event is suggestive of a deletion,

translocation or repeat expansion/contraction. Note that in all examples, these reads are rare and our pipeline does not use the same levels of support required by most SV callers. Examples are snapshots from IGV.

2. Fig 2A: is the difference between "young" and "old" statistically significant?

Reply: This difference is at best marginally significant (see reply to Comment 1 of Reviewer 2). We have specified this in the revised manuscript.

3. The authors should discuss what is the origin of the transcript downstream of tlh2 in their opinion. Is it transcribed by RNA Polymerase II? Where does it originate from exactly?

Reply: We can only speculate about the nature of these transcripts at this point, but now discuss that they might be small non-coding transcripts involved in chromatin regulation/silencing in this sub-telomeric region. As mentioned above, their expression might be triggered by a small up-regulation of *tlh2* transcripts in ageing cells.

4. Fig S4 seems a little bit out of place here, considering that the authors never pursue the idea that transcription factors could trigger DSBs and microhomology-mediated rearrangements. And it is a clearly distinct hypothesis from their R-loop hypothesis.

Reply: We agree that this is outside the main focus of the manuscript (hence in supplement). But given the indications that R-loops cannot explain everything, we still consider these to be valuable observations that could guide future work into mechanisms. See also our response to Comment 4 of Reviewer 2.

Reviewer #2:

*****Major comments:*****

1. Analysis of human brain cells: I was not fully convinced by this analysis that shows very subtle differences between young and old donors that are not statistically analyzed (panel A). I would like to see p values here. I was also wondering whether the young brains may not already be too old, knowing that this tissue is experiencing a lot of oxidative stress in humans. If possible, and if data are available, it would be interesting to see the same type of analysis brains from newborn and old mice for instance. Alternatively, could the same type of analysis be performed in other types of human tissues to strengthen the conclusions?

Reply: We agree that the difference between young and old human donors is quite subtle, but the coverage and sample number in this data set were low (Methods). We also agree that the younger (adult) brains in our analyses will have already experienced age-associated physiological decline. However, the most striking result from this analysis was not the difference between old and young, but the distribution in microhomology usage and the parallels to our model yeast system (albeit with an interesting bump in the part of the distribution associated with longer microhomology usage which, as we discuss, may be indicative of differences in break repair in human brain and a greater reliance on single-strand annealing). Regarding the difference between young and old human donors, we have applied various statistical tests to the data, suggesting that the difference is marginally significant at best: comparison of number of junctions per mapped read (left below; $p_{Mann\text{-}Whitney} = 0.23$); correlation between sample age and JPMR (right below; $p_{Pearson} = 0.17$); comparison of

average microhomology length between pooled old and young samples ($p_{T\text{-test}} = 0.05$). We are cautiously discussing these data in the revised manuscript.

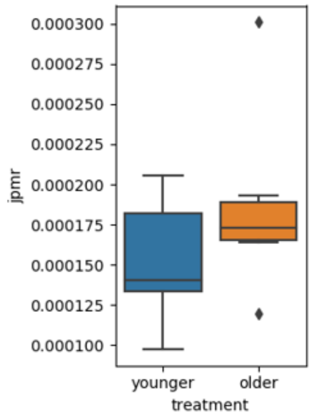

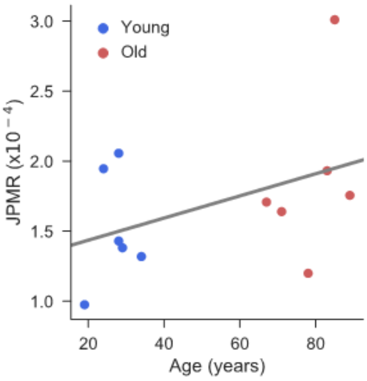

As mentioned by the Reviewer, this small difference could reflect that brain cells even from younger adults are already aged to some extent. In addition, the available data are quite noisy and sparse. We searched the ENA repository for alternative data as suggested by the Reviewer but could not find any from appropriate cell types (e.g., not blood) or with associated metadata describing the age. Zhang et al. (2019) have reported DNA alterations during ageing using single-cell sequencing data, which we cite in the revised manuscript.

2. The link between sir2 and tlh2 in the text (Page 10) is not easy to follow. Does sir2 expression and/or activity decrease in ageing fission yeast cells that may account for the increase in tlh2 RNA levels in ageing cells? Authors should also check the levels of RNA:DNA hybrids downstream of tlh2 locus in sir2-deleted cells to strengthen the connection between sir2 and tlh2.

Reply: The expression of *sir2* does not decrease in ageing cells based on our RNA-seq data; if anything, it slightly increases (shown at right). Intriguingly, however, Sir2 in budding yeast increases the mutation frequency specifically in ageing cells [Fabrizio et al. *Cell* (2005) 123: 655]. We have now clarified and better discussed this section in the revised manuscript.

As mentioned in reply to Reviewer 1 above, to obtain better insights into the role of Sir2 to generate rearrangements, we are determining the lifespan of *sir2* deletion mutants and will sequence PCR amplicons of the *tlh2* region in *sir2* mutant and wild-type control cells as a function of age. If successful, these experiments would show that Sir2 is required for rearrangements in selected hotspots.

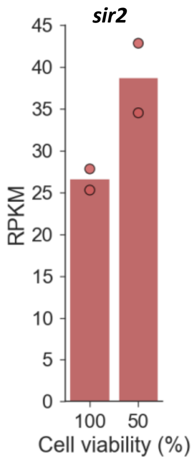

3. The authors convincingly show that scw1-deleted cells show higher levels of R-loops and suggest that the age-dependent decrease of Scw1 protein levels may thus account for the R-loop increase in ageing cells. This, however, remains correlative. It would be interesting to overexpress Scw1 in aged cells to see whether this reduces R-loops. This, however, implies that Scw1 protein does not aggregate in ageing cells.

Reply: We agree that our findings are largely correlative at this point. The budding yeast orthologue of Scw1, Whi3, aggregates during ageing (Schlissel et al. 2017, cited in manuscript). Moreover, according to the published ORFeome data for fission yeast, Scw1 forms aggregates when overexpressed (see figure below, showing Scw1-YFP signal) [Matsuyama et al. *Nat Biotechnol* (2006)

24:841]. Thus, we do not think that an overexpression experiment will be conclusive. We now mention this in the revised manuscript.

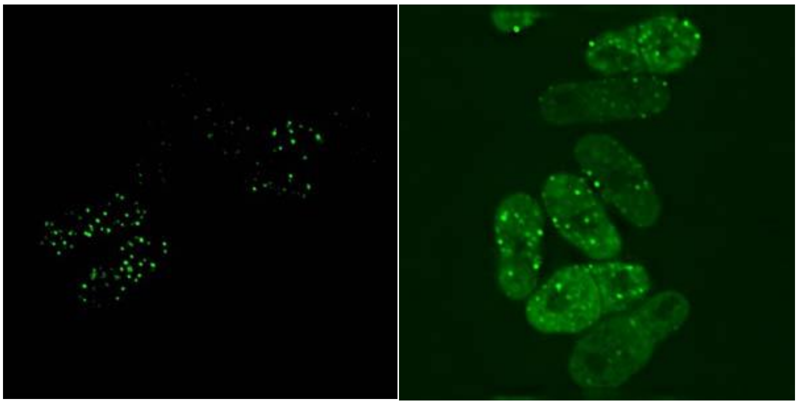

4. I am slightly confused by the analysis of motifs at microhomology-mediated junctions. Fig S4 suggests that many of these motifs may correspond to transcription factor binding sites. Did I understand properly that these analyzed regions corresponded to all junctions, including those located at 3' UTR regions? If so, how does this observation fit with the analysis of page 11 that reports on the enrichment for Scw1 targets? What is the motif for Scw1 binding? Does it match to any of the motifs identified in Fig S4? This should be clarified.

Reply: A similar point has been brought up by Reviewer 1 (Minor point 4). This analysis is outside the main focus of the manuscript and not necessarily linked to Scw1. It was based on a broader, unbiased analysis or all microhomology-containing junctions. But given the indications that R-loops cannot explain everything, we still think that these are useful observations that could guide future work into mechanisms. The motif for Scw1 has been predicted informatically. Its absence from our motif analysis probably reflects the specific questions we were asking and subsets of data that were being used (Scw1 came out of an analysis specifically looking for genes with more junctions in their 3' UTR than would be expected by chance). In the revised manuscript, we now better highlight that mechanisms other than R-loops may contribute to the observed rearrangements, including transcription.

*****Minor comment/suggestion:*****

- I was wondering whether there may be any link between R-loops and the microhomology-mediated end-joining mechanism? In human cells, a recent screen identified a large set of proteins binding to RNA:DNA hybrids (Wang et al, Genome Res 2018). Interestingly, LIG3 was among them. Knowing that this ligase has been involved in that specific DNA repair pathway, this may be interesting to speculate about this for fission yeast.

Reply: Thank you for pointing to this reference. We have now added this as a discussion point to the revised manuscript.
