# Supplementary material for: R-loops and regulatory changes in chronologically ageing fission yeast cells drive non-random patterns of genome rearrangements
Source: PLoS Genet. 2021 Aug 31;17(8):e1009784. doi: 10.1371/journal.pgen.1009784 (PMC8437301; doi:10.1371/journal.pgen.1009784)
Supplement: Attachment — Submitted filename: Rebuttal2.docx [file pgen.1009784.s017.docx]

We thank the two Reviewers for their additional Comments, which are addressed point-by-point below.

**Comments to the Authors:
Please note here if the review is uploaded as an attachment.**

Reviewer #1: Ellis et al., present a really exciting and novel link between chronological aging, changes to RNA binding proteins and associated accumulation of R-loop linked DNA rearrangements. I appreciated the thoughtful revisions and rebuttal letter that the authors prepared for the previous reviewers. Overall, they seem to have addressed most of the concerns and have significantly improved the story. Here are a couple of points to consider clarifying in the manuscript.

- I appreciate that the authors have tempered their language comparing young and old brains in Figure 2. I would encourage them to be explicit in their language similar to the rebuttal letter. Basically stating the differences are marginally significant at best, and the really important finding is the same pattern of rearrangements in non-dividing somatic cells (or any age) and the chronologically aged S. pombe.

Reply: On page 7, we now more explicitly describe the data as suggested by the Reviewer: New text: “We found a subtle increase in junctions in older brain cells (Fig. 2A), although differences were marginally significant at best, reflecting that the coverage and sample number in this data set were low (Methods). Note that the younger brains in this analysis will have already experienced some level of age-associated physiological decline. The most striking result from this analysis was the similar pattern of rearrangements: as in fission yeast (Fig. 1D), the junctions were associated with microhomology in somatic human cells (Fig. 2A).”

- On page 11 the authors cite Wang et al., to state that MMEJ proteins have been proposed to bind RNA:DNA hybrids. Which proteins, and are they conserved in S. pombe should be stated.

Reply: We now specify on p. 11 that the LIG3 DNA ligase identified in the screen is involved in MMEJ, which does not appear to be conserved in *S pombe*.

- On page 12/13 the identification of the Scw1 orthologues RBPMS and RBPMS2 in the search for the WRN gene is cited as somehow meaningful. I did not understand why this was included, surely this is a coincidence of mapping the WRN gene to a large region that happened to contain an RBPMS? Unless I am missing some context I would delete this statement.

Reply: We agree and have now deleted that statement.

- The Tlh1 hotspot is important, as are Scw1 target genes, but the authors do not tell us if the 3’ transcript at Tlh1 is a Scw1 target gene. Surely this can be extracted from their datasets and it should be mentioned one way or the other.

Reply: The transcript downstream of *tlh2* is not annotated as a gene and is not among the Scw1 targets (neither is *tlh2*). Note that we implicate *tlh2* and Scw1 in different types of hotspots (global vs local), so they would not be expected to act together.

- The author acknowledge that the excess Tlh1 protein could be playing some role, since it's a RecQ helicase, but do not really explore this. Could Tlh1 protein overexpression, for example from a plasmid, cause a dominant genome instability phenotype and lead to rearrangements?

Reply: We do show *tlh2* overexpression data (Fig. S7), albeit not from a plasmid, and suggest that it causes rearrangements owing to transcriptional stress or via the Tlh2 protein itself. It may well be worth to further explore any direct involvement of Tlh2 in future work.

- I would make a point of citing the recent Chedin paper on S9.6 and highlighting the RNaseIII data, and the DRIP-seq data (which is less prone to artefacts), as a way to strengthen the conclusions. The immunofluorescence data with S9.6 cannot stand alone, but the authors have addressed this, they should make it clear to readers.

Reply: As suggested, we now highlight these control data more explicitly on page 13 to strengthen the conclusion.

- Finally, it would be nice to hear more speculation about where the breaks come from at the tlh1 or other hot spots. In non-dividing cells replication fork collisions cannot be the driver. Sordet and Gromak had a paper a few years ago (PMID: 31533039) showing how nucleases and Topoisomerases can drive breaks at these sites. It would be worth citing and discussing whether you think something similar is happening.

Reply: Thank you for pointing to this relevant paper. We have now added this reference and speculation on page 15.

Reviewer #2: No attachment.
On reading the rebuttal letter and the two versions of the manuscript I was able to appreciate the efforts and the quality of the improvements made in the revised version of the manuscript from Bahler and co-workers.

Considering the quality and originality of the initial observation, the wide array of methods accessible in yeast this work was positioned to generate solid and interesting results on DNA repair in ageing (stationary phase) cells. The sequencing and analysis methods used approach the very best in functional genomics, that was further improved in the revised version of the manuscript. The results are analyzed with a certain respect for possible caveats, which were rightly raised by the reviewers. The remarks of the reviewers in particular about hybrid RNA-DNAs allowed a relevant analysis of the mutants sir2, rnh1 and rnh201. The new results in the second version of the manuscript have made it possible to move from the description stage to the process stage allowing a model in which a subset of R-loops is favored in the absence of Scw1 and targets genome rearrangements. Personally, I am not a fan of this model, but it agrees with the results offered here.

Taken as a whole the new version of the manuscript is satisfactory for publication. However, I find the discussion genetically disappointing here, with the lack of discussion of the break-induce replication (BIR) process, Dicer's role in telomeres (tlh1-4) and rDNA (especially in fission yeast), topoisomerase 1, and more generally the organization of chromosomes in aging cells.

Reply: We have added more discussion on p. 15 with respect to topoisomerase I and endonucleases as suggested by Reviewer #1. A discussion about Dicer’s role in telomeres and rDNA, however, seemed too speculative and remote from the focus of this study. Given that the manuscript already cites 127 references, we did not further extend the discussion in this direction.
